# Self-Reported Voluntary Blame-Taking: Kinship Before Friendship and No Effect of Incentives

**DOI:** 10.3389/fpsyg.2021.621960

**Published:** 2021-02-02

**Authors:** Teresa Schneider, Melanie Sauerland, Harald Merckelbach, Jens Puschke, J. Christopher Cohrs

**Affiliations:** ^1^Department of Law, Institute of Criminal Sciences, Philipps-University Marburg, Marburg, Germany; ^2^Section of Social Psychology, Department of Psychology, Philipps-University Marburg, Marburg, Germany; ^3^Section of Forensic Psychology, Department of Clinical Psychological Science, Faculty of Psychology and Neuroscience, Maastricht University, Maastricht, Netherlands

**Keywords:** close relationship, *Kinship Premium*, prosocial behavior, *Social Exchange Theory*, voluntary false confessions

## Abstract

Inspired by theories of prosocial behavior, we tested the effect of relationship status and incentives on intended voluntary blame-taking in two experiments (Experiment 2 was pre-registered). Participants (*N*_E1_ = 211 and *N*_E2_ = 232) imagined a close family member, a close friend, or an acquaintance and read a scenario that described this person committing a minor traffic offense. The person offered either a monetary, social, or no incentive for taking the blame. Participants indicated their willingness to take the blame and reasons for and against blame-taking. Overall, a sizable proportion of participants indicated to be willing to take the blame (E_1_: 57.8%; E_2_: 34.9%). Blame-taking rates were higher for family members than close friends or acquaintances in both experiments, as expected. Unexpectedly, there was no difference between a close friend and an acquaintance in Experiment 2. Social incentives did not have an effect on voluntary blame-taking in either experiment. Neither did we find an interaction between relationship status and incentives. The results highlight the importance of kin relationships in the context of voluntary blame-taking.

## Introduction

In 2002, Günther Kaufmann, a well-known German actor, was sentenced to 15 years in prison. He had confessed to murdering his friend and tax accountant. In a retrial 3 years later, Kaufmann was acquitted after the real perpetrators, his wife Alexandra, her previous lover, and two accomplices, had been identified. It became clear that Kaufmann confessed to the murder to protect his terminally ill wife who died in the course of the trial (Otto, 2006). This case is not an exception and individual cases show that voluntary blame-taking can occur for serious crimes. Furthermore, self-report studies suggest that voluntary blame-taking for minor offenses to protect friends, partners, or family members occurs even more often than coerced false confessions (e.g., Sigurdsson and Gudjonsson, 1996; Gudjonsson et al., 2007; Willard et al., 2015; Volbert et al., 2019). Although a lot of research has identified risk factors for coerced false confessions, voluntary false confessions to protect someone else are often overlooked. To our knowledge, only four experimental studies have been published in this area (Pimentel et al., 2015; Willard et al., 2016; Willard and Burger, 2018; Schneider et al., 2020). With this in mind, the current study investigates the situational risk factors *relationship status* and *incentives*, using theories of prosocial behavior as a theoretical background.

In contrast to coerced false confessions, voluntary false confessions are defined as self-incriminating statements offered without external pressure from the police (Kassin and Wrightsman, 1985). As underlying reasons, a pathological need for fame, desire to be recognized, mental illness, delusional thinking, excessive feelings of guilt, or the desire to protect someone else have been suggested (McCann, 1998). Here, we will focus on voluntary false confessions to protect someone else, a phenomenon that we refer to as voluntary blame-taking.

It is difficult to establish how often voluntary blame-taking occurs. In such cases, confessors have little or no motivation to uncover the injustice, either because sanctions are relatively mild (e.g., a probation sentence, short incarceration; Redlich et al., 2010), or because the actual culprit is important to them and they still want to protect them (Gudjonsson and Sigurdsson, 1994). In an analysis of 1,110 retrials in Germany, voluntary false confessions to protect the real perpetrator occurred in 22 of 66 false confession cases (Lange, 1980). One of the most common reasons mentioned in self-report studies is to *protect somebody else* (Sigurdsson and Gudjonsson, 1996; Gudjonsson et al., 2007, 2019; Willard et al., 2015; Volbert et al., 2019). To understand this behavior, theories of prosocial behavior can provide a helpful framework.

### Kinship, Close Relationship, and Prosocial Behavior

One factor that influences prosocial behavior is the degree of relatedness and the relationship between the helper and the person in need. *Kin Selection Theory* proposes that evolutionary mechanisms motivate people to help others according to their degree of relatedness because they want to ensure the survival of their genes (Hamilton, 1964). In line with this theory, people are more likely to help kin (Burnstein et al., 1994; Madsen et al., 2007) and particularly so in life-and-death or high-cost help situations (Stewart-Williams, 2007). However, a different research line suggests that emotional closeness rather than genetic relatedness promotes helping behavior among kin and non-kin, and therefore, also blame-taking (*Close Relationship Model*; Korchmaros and Kenny, 2006). Support for the idea that emotional closeness guides helping behavior comes from research showing that people send more money to close friends than strangers (Leider et al., 2009) and that relationship closeness mediates the effect of close friendship on the amount of money sent (Hackman et al., 2015).

Other studies suggest that emotional closeness is not sufficient when explaining prosocial behavior among close relatives and friends. Here, participants favor kin over non-kin at the same level of emotional closeness (*Kinship Premium*; Rachlin and Jones, 2008; Curry et al., 2013; Pollet et al., 2013; Hackman et al., 2015). Indeed, people seem to use cues other than emotional closeness when helping kin, whereas emotional closeness might be the key mediator for increased helping among friends (Hackman et al., 2015).

In the context of blame-taking, 61% of college students who had previously been in a blame-taking situation took the blame for someone else in that situation and reported they had done so for a friend or partner (67%; Willard et al., 2015). In another college student sample, 70% reported taking the blame for a friend (Gudjonsson et al., 2007). In a substance abuse treatment sample, non-trivial proportions of blame-takers reported taking the blame for a friend or partner (39%) or a family member (32%; Willard et al., 2015). These findings suggest that the relationship between the confessor and the real perpetrator is important when studying voluntary blame-taking.

So far, two experimental studies have directly examined the influence of relationship closeness on intended voluntary blame-taking. In both studies, participants thought about a close or a casual friend and, guided by a scenario, imagined this person committing a transgression (Willard et al., 2016; Willard and Burger, 2018). Participants were more willing to take the blame for a close than a casual friend. However, neither study tested possible differences between friends and family members. In the current line of research, we tested whether there is a difference in the likelihood of voluntary blame-taking for a close family member, a close friend, or an acquaintance. Opposing predictions can be derived from the *Close Relationship Model* and *Kinship Premium*. The *Close Relationship Model* predicts similar blame-taking rates for close family members and close friends and lower rates for acquaintances (Hypothesis 1a). *Kinship Premium*, on the other hand, predicts higher blame-taking rates for close family members than close friends at the same level of emotional closeness and higher blame-taking rates for close family members or close friends than acquaintances (Hypothesis 1b).

### Incentives and Prosocial Behavior

Another theory that explains prosocial behavior is *Social Exchange Theory*, which holds that prosocial behavior is based on self-interest and that people estimate costs and benefits before helping another person (Homans, 1961; Foa and Foa, 1976). More specifically, people are more likely to help others when the benefits (e.g., increased self-esteem and money) exceed the costs (e.g., embarrassment, pain, and time). Accordingly, incentives should increase prosocial behavior.

Research on the link between incentives and prosocial behavior suggests that the kind and size of the incentive are crucial. Monetary incentives in particular can have opposing effects when it comes to prosocial behavior (Gneezy et al., 2011). For example, small monetary incentives decrease the amount of money voluntarily collected for a charity compared with no incentive, whereas a large monetary incentive did not differ from a no-incentive condition (Gneezy and Rustichini, 2000). Furthermore, monetary incentives can increase prosocial behavior in private but not in public (Ariely et al., 2009).

Large-scale field experiments in collaboration with the Red Cross showed that non-monetary incentives such as lottery tickets and gift cards had positive effects on the number of donors (Goette and Stutzer, 2008; Lacetera et al., 2014). In one study offering different kinds of incentives, participants rated how likely other students would be to help load a sofa into a van (Heyman and Ariely, 2004). They were offered either cash, candy, or “monetized candy” (the cost of the candy was mentioned) at a low ($0.50) or medium payment level ($5.00). Another group received no payment. The expected willingness to help in the low-payment level of the cash and “monetized candy” condition was below that in the no-payment and the candy condition. Furthermore, willingness to help increased when payment level increased in the cash condition but not in the candy condition. The authors argued that monetary incentives can change the framing of a situation and diminish the perception of the interaction as social. As a result, small monetary incentives can negatively affect prosocial behavior, but social incentives can be close substitutes for money without negatively affecting prosocial behavior.

Combined, these findings suggest that people display more effort for a good cause when they are able to signal the effort to others and that a monetary award in public can crowd out prosocial behavior due to *image motivation* (Gneezy et al., 2011). In the context of blame-taking, tentative support exists for the idea that incentives foster prosocial behavior. In a survey with a substance abuse treatment sample, self-reported blame-takers reported being offered more incentives to take the blame than those who did not take the blame (Willard et al., 2015). Drawing from these results, we expected that people who were offered a social incentive would be more willing to take the blame than people who were offered a monetary incentive or no incentive (Hypothesis 2).

### Interaction Between Relationship Status and Incentives

Another theory suggests that there is a different motivation behind prosocial behavior depending on type of relationship. *The Empathy-Altruism Hypothesis* holds that people show altruistic behavior when they feel empathy toward somebody else. When they do not feel empathy, people estimate costs and benefits before helping as proposed in *Social Exchange Theory* (Batson, 1991). Empirical work suggests that cost-benefit analyses could play an important role in exchange relationships (e.g., acquaintance) but not in communal relationships (e.g., friendships, romantic relationships, and family relationships; Clark and Mills, 1993). For example, friends complied more with a request to purchase raffle tickets than strangers regardless of a pregiving favor. Among strangers, a pre-giving favor (a soda) elicited more compliance than a direct request (Boster et al., 1995). We, therefore, expected an interaction between relationship status and incentives. Specially, we expected that incentives more strongly promote the intended blame-taking rate in the acquaintance condition compared with the family member or close friend condition (Hypothesis 3).

### Overview of the Experiments

We conducted two experiments that tested the effect of relationship status and incentives on intended voluntary blame-taking. Following earlier work (Willard et al., 2016; Willard and Burger, 2018), participants thought about a close family member, a close friend, or an acquaintance and imagined that this person had committed a traffic offense: the person drove 36 km/h too fast and is now facing an entry in the drivers’ registry for traffic violations and a fine of €120. The person committed traffic offenses on several occasions in the past and her/his driving license would be revoked if she/he assumed guilt. The person offered the participant a monetary, social, or no incentive for taking the blame. In Experiment 1, the person promised to talk about the good deed in a circle of friends/the family/acquaintances (social incentive), or offered to pay €50 for taking the blame (monetary incentive). We asked participants whether they would take the blame or not and why.

In response to null findings for the incentives manipulation in Experiment 1, we modified the social incentive condition in the pre-registered Experiment 2. Here, the person offered the participant to cook her/his favored meal as a thank-you. Furthermore, we increased the monetary incentive to €400 and explicitly stated that it was offered in private to test whether more money would increase the blame-taking rate (Ariely et al., 2009).

## Materials and Methods

### Participants

Across both experiments, we tested 535 participants online using the website SoSci Survey (Leiner, 2019). We excluded participants when they indicated that the person imagined would never ask them to take the blame (*n_E1_* = 14; *n_E2_* = 3), when they did not describe a person when prompted (*n_E1_* = 5; *n_E2_* = 45), and when they described a romantic partner (*n_E1_* = 3; *n_E2_* = 22). In Experiment 1, we tested participants (*N* = 211; 155 female, 1 missing value; *M_age_* = 26.01 years, *SD_age_* = 10.36) in exchange for 0.5 participation credit or without compensation. Participants were mostly students (77.6%) or employees (13.8%). The majority had a high school degree (62.6%).

In Experiment 2, we tested participants (*N* = 232; 126 female; *M_age_* = 49.97 years, *SD_age_* = 14.91) through the online panel *respondi*.^1^ Participants were mostly employees (52.8%) and only 2.2% were students. The majority had either a completed vocational training (24.7%), a secondary school diploma (23.3%), or a high school degree (14.2%). Participants received €0.60 for participating (Participants needed 12 min to complete the online study. The compensation was determined by the panel company; 0.05€/min). We preregistered Experiment 2 at the Open Science Framework using the template form “AsPredicted.org” (https://osf.io/g6jch).

### Design

In both Experiments, we assigned participants randomly in a 3 (relationship status: *close family member* vs. *close friend* vs. *acquaintance*) x 3 (incentive: *social* vs. *monetary* vs. *none*) between-participants design. In Experiment 1, we also attempted to manipulate mood as a third factor (*negative* vs. *positive* vs. *neutral*) by means of the scale *creativity* from *the Berlin Intelligence Structure Test* (Jäger et al., 1997), but the manipulation check suggested that the manipulation was not successful. We, therefore, do not discuss this factor further. Blame-taking was our outcome variable. We were also interested in participants’ reasons for (not) taking the blame.

To manipulate relationship status, we gave participants a description of either a close family member, a close friend, or an acquaintance and asked them to imagine the described person (E_1_: *n*_close family_ = 70, *n*_close friend_ = 74, *n*_aquaintance_ = 67; E_2_: *n*_close family_ = 68, *n*_close friend_ = 82, *n*_aquaintance_ = 82). The incentives varied across the two experiments. In Experiment 1, the social incentive constituted the promise to talk about the good deed in a circle of friends/the family/acquaintances. In Experiment 2, the person offered the participant to cook her/his favored meal (E_1_: *n*_social incentive_ = 71, *n*_monetary incentive_ = 71, *n*_no incentive_ = 69; E_2_: *n*_social incentive_ = 77, *n*_monetary incentive_ = 76, *n*_no incentive_ = 79). The monetary incentive concerned the payment of €50 (Experiment 1) or €400 (Experiment 2), respectively. In Experiment 2, we asked participants whether the person offered them something for taking the blame and if so, what. Most of them (70.7%; *n* = 164) correctly identified the type of incentive offered. We excluded the remaining participants (*n* = 68) from the analysis examining the influence of incentives on blame-taking. However, this did not affect the pattern of results.

### Materials

#### Relationship Status Manipulation

Participants in the close family member condition read the following description:

*In this case a related person is a close family member (e.g., father, mother, brother, sister, …) who is over 16 years old. You have a trusting relationship with each other and are positive about each other*.

Participants in the close friend condition read the following description:

A friend is someone you have known for at least a year and to whom you are not related. You have a trusting relationship and feel comfortable sharing personal information. You know each other very well and would be there for each other in difficult times. (A friend is not someone with whom you have a romantic relationship.)

Participants in the acquaintance condition read the following description:

*An acquaintance is a person with whom you have not spoken much more than once and whom you do not see very often* (in Experiment 2, we changed it to *with whom you have not spoken often* instead of *with whom you have not spoken much more than once). However, you should not have any dislike or special affection for the person. You do not have a trusting relationship with the person and would not communicate any personal concerns to the person. You do not know each other very well*.

Afterward, participants reported some background information about the person and the relationship.

#### Relationship Status Manipulation Check

We formulated eight relationship closeness items to serve as a manipulation check for the relationship status manipulation. Example statements are *I like that person very much* or *I think about that person a lot*. Item were rated on a scale from 1 (*do not agree at all*) to 7 (*fully agree*). We added those items up to create a composite variable as a measure of relationship closeness. The variable had excellent internal consistency (Cronbach’s *α*_E1_ = 0.97 and *α*_E2_ = 0.96).

#### Plausibility of Scenario

To check the plausibility of the scenario’s realism and seriousness as well as the seriousness of the consequences, participants rated the scenario with five questions on a scale from 1 (*do not agree at all*) to 5 (*fully agree*). On average participants indicated that they were able to imagine the situation well (E_1_: *M* = 3.89, *SD* = 1.13; E_2_: *M* = 4.09, *SD* = 1.19). Participants rated the scenario’s realism (E_1_: *M* = 2.97, *SD* = 1.36; E_2_: *M* = 3.23, *SD* = 1.39), seriousness (E_1_: *M* = 2.60, *SD* = 1.03; E_2_: *M* = 3.10, *SD* = 1.17), and the seriousness of the consequences (E_1_: *M* = 2.54, *SD* = 1.25; E_2_: *M* = 2.72, *SD* = 1.31) as moderate to high. Participants rated their ability to imagine that such a situation could happen to them with the imagined person as relatively low (E_1_: *M* = 1.94, *SD* = 1.30; E_2_: *M* = 2.01, *SD* = 1.25).

#### Reasons for and Against Blame-Taking

Blame-takers indicated their reasons for blame-taking by selecting one or more of the following options: (a) *the reward the person promised me convinced me*; (b) *I took the blame because I think I am a good person*; (c) *I know the other person well*; (d) *I like that person*; (e) *I felt sorry for that person*; (f) *the person had greater consequences to fear than I did*; (g) *the person would do the same for me*; (h) *I took the blame because I felt pressured*; (i) *I took the blame because I was scared*; (j) *I owed the other person something*; (k) *other*. In Experiment 2, we added (l) *the person is important to me*, (m) *I can ask the person for a favor now*, and (n) *I wanted to help the other person*.

Participants who were reluctant to take the blame (i.e., non-blame-takers) indicated their reasons by selecting from the following options: (a) *that person was not important to me*; (b) *the reward was not big enough*; (c) *I am an honest person*; (d) *it was the person’s own fault*; (e) *the person would not do the same for me*; (f) *I felt too pressured*; (g) *I was too scared*; (h) *other*. In Experiment 2, we added (i) *the consequences were too serious*, (j) *the other person would not learn from her/his mistake*, and (k) *it is against the law*.

### Procedure

Participants provided consent and demographic information (In Experiment 1, participants completed a creativity test and were randomly assigned to receive fictional positive, negative, or no feedback before imagining a person. Furthermore, they answered the mood manipulation check after the creativity test and again at the end of the experiment). Participants then thought about either a close family member, a close friend, or an acquaintance who fit the description and provided background information about this person. Participants then had to imagine that this person drove 36 km/h too fast a few months ago and was now facing an entry in the drivers’ registry for traffic violations and a fine of €120. The person committed traffic offenses on several occasions in the past and her/his driving license would be revoked if she/he assumed guilt. The person makes it clear that it is really urgent, because otherwise she/he would be compromised in everyday life. The person knows that the participant does not have an entry yet and has never attracted attention in traffic. Therefore, she/he desperately asks the participant to take the blame. Due to the limited quality of the pertinent driver picture produced by the speed control device, it would be very likely that the statement would be believed. The fine would be refunded by the person asking the favor, but the participant would have to accept an entry at the drivers’ registry for traffic violations. The person offered a monetary (E_1_: €50/E_2_: €400), social (E_1_: the person will talk about the good deed in a circle of friends/the family/acquaintances/E_2_: the person offered the participant to cook her/his favored meal), or no incentive for taking the blame. After making a decision to (not) take the blame, participants indicated whether they had experienced a similar situation before and if so whether they had taken the blame and provided reasons for falsely taking the blame. Finally, participants answered the scenario plausibility questions and received the debriefing.

## Results

For Experiment 1, we conducted a *post hoc* sensitivity analysis using GPower (Faul et al., 2007) with a power of 0.80, *α* = 0.05, and *N* = 211 because our *a priori* analysis included three factors (*relationship*, *incentive*, and *mood*). It revealed a detectable effect size of *ϕ* = 0.21 (i.e., an effect size corresponding to a Cohen’s *d* of 0.43). One study that tested the influence or relationship closeness on blame-taking found a small to moderate effect size of *d* = 0.40 (Willard et al., 2016). For Experiment 2, we conducted an *a priori* power analysis using GPower with a power of 0.80, *α* = 0.05, and a medium effect size of *ϕ* = 0.25 that resulted in an aspired sample size of 241.

### Data Analyses

We conducted univariate ANOVAs to test whether the manipulation of relationship status was successful. To test the influence of relationship status and incentive on the blame-taking rate, we conducted logistic regressions for both experiments with relationship status (reference category = close friend) and incentive (reference category = social incentive) as predictors. We also conducted separate chi-square tests for each factor. Because the results were analogous to the logistic regressions, we report these less complex analyses and used the Bonferroni correction to correct for multiple comparisons. To evaluate the interaction of relationship status and incentive, we carried out a logistic regression.

### Manipulation Check Relationship Status

In both experiments, a univariate ANOVA showed a significant main effect of relationship status (close family member vs. close friend vs. acquaintance) on relationship closeness [E_1_: *F*(2, 208) = 477.82, *p* < 0.001, *η*^2^ = 0.82; E_2_: *F*(2, 229) = 137.14, *p* < 0.001, *η*^2^ = 0.54]. As expected, *post hoc* tests using the Bonferroni correction indicated that the close family member condition (E_1_: *M* = 48.06, *SD* = 6.64; E_2_: *M* = 46.18, *SD* = 8.81) did not differ significantly from the close friend condition (E_1_: *M* = 47.86, *SD* = 5.97; E_2_: *M* = 44.50, *SD* = 8.20), *ps* ≥ 0.886. We did not expect a difference because in both conditions, participants thought about a person with whom they had a trusting relationship. Also, as expected, both the close family member and the close friend conditions differed significantly from the acquaintance condition (E_1_: *M* = 17.15, *SD* = 7.58; E_2_: *M* = 23.13, *SD* = 11.70), *ps* < 0.001. These results indicate that our relationship manipulation was successful.

### Planned Analyses: Effect of Relationship Status and Incentives on Voluntary Blame-Taking

Table 1 shows the frequency and percentage of intended blame-taking across both experiments. In Experiment 1, 122 of the 211 (57.8%) participants indicated that they would take the blame for the driver’s violation in the scenario; in Experiment 2, 81 of 232 participants (34.9%) did so. Participants who indicated that something similar had happened to them in real life, took the blame in 60.7% (17 of 28; Experiment 1) and 46.7% of the cases (7 of 15; Experiment 2), respectively.

**Table 1:** Frequency and proportion of voluntary blame-taking in Experiments 1 and 2.

	Blame-taking rates	
	Experiment 1 (*N* = 211)	Experiment 2 (*N* = 232)
Relationship status	*n* (%)	*n* (%)
Close family member	59 (84.3)^a^	36 (52.9)^d^
Close friend	47 (63.5)^b^	26 (31.7)^e^
Acquaintance	16 (23.9)^c^	19 (23.2)^e^
Across conditions	122 (57.8)	81 (34.9)
**Incentives**	**Experiment 1 (*N* = 211)**	**Experiment 2 (*n* = 164)**
Social incentive	43 (60.6)	16 (35.6)
Financial incentive	41 (57.7)	19 (38.0)
No incentive	38 (55.1)	26 (37.7)
Across conditions	122 (57.8)	61 (37.2)

^a-e^Within each column, proportions with different superscript letter are significantly different at the *p* = 0.016 level.

Hypothesis 1a predicted similar blame-taking rates for close family members and close friends and higher blame-taking rates for close family members and close friends than for acquaintances. Hypothesis 1b predicted higher blame-taking rates for close family members than close friends and higher blame-taking rates for close family members or close friends than acquaintances. In Experiment 1, blame-taking differed as a function of relationship status, *χ*^2^(2, *N* = 211) = 52.73, *p* < 0.001, *ϕ* = 0.50. Participants took the blame in 84.3% (close family member condition), 63.5% (close friend condition), and 23.9% (acquaintance condition) of the cases. Supporting Hypothesis 1b, all three conditions differed significantly from each other with large effect sizes for the comparisons between the acquaintance and close friend conditions, *χ*^2^(1, *N* = 141) = 22.35, *p* < 0.001, *ϕ* = 0.40, and the acquaintance and close family member conditions, *χ*^2^(1, *N* = 137) = 50.42, *p* < 0.001, *ϕ* = 0.61. We found a small effect for the comparison between the close family member and close friend conditions, *χ*^2^(1, *N* = 144) = 7.99, *p* = 0.005, *ϕ* = 0.24.

In Experiment 2, blame-taking also differed as a function of relationship status, *χ*^2^(2, *N* = 232) = 15.07, *p* = 0.001, *ϕ* = 0.26. Participants took the blame in 52.9% (close family member condition), 31.7% (close friend condition), and 23.2% (acquaintance condition) of the cases. As predicted by Hypothesis 1b, the close family member condition differed significantly from the close friend condition, *χ*^2^(1, *N* = 150) = 6.91, *p* = 0.009, *ϕ* = 0.22, and the acquaintance condition, *χ*^2^(1, *N* = 150) = 14.19, *p* < 0.001, *ϕ* = 0.31. The effect sizes were small to moderate. In contrast to what would be expected on the basis of Hypotheses 1a and 1b, there was no significant difference between the close friend and the acquaintance conditions, *χ*^2^(1, *N* = 164) = 1.50, *p* = 0.221, *ϕ* = 0.10.

Hypothesis 2 predicted that participants who were offered a social incentive would be more willing to take the blame than those who were offered a monetary or no incentive. Unexpectedly, blame-taking did not differ as a function of incentive in either experiment, *χ*^2^(2, *Ns* = 211/164) ≤ 0.43, *ps* ≥ 0.805, *ϕs* ≤ 0.05. In the social incentive condition, 60.6% (E_2_: 35.6%) of participants took the blame, in the monetary incentive condition 57.7% (E_2_: 38.0%), and in the no incentive condition 55.1% (E2: 37.7%).

Hypothesis 3 predicted that incentives have a stronger effect in the acquaintance condition than in the family member or close friend condition. The overall Wald statistic for the interaction was non-significant in both experiments, Wald *χ*^2^(2, *Ns* = 211 and 164, respectively) = 2.67 and 4.23, *ps* = 0.615 and 0.376, respectively.

### Exploratory Analyses: Reasons for and Against Voluntary Blame-Taking

Tables 2 and 3 show participants’ reasons for and against blame-taking. In Experiment 1, most blame-takers indicated taking the blame because *the other person had greater consequences to fear* (85.2%), *they liked the other person* (77.9%), *the other person would do the same for them* (65.6%), and *they knew the other person well* (63.9%). Similarly, in Experiment 2, most blame-takers indicated that they took the blame because *the other person was important to them* (70.4%), *the other person had greater consequences to fear* (69.1%), *they liked the other person* (58.0%), *they wanted to help the other person* (55.6%), and *the other person would do the same for them* (54.3%). In either experiment, only one person indicated *the reward convinced her/him* (E_1_: 0.2%; E_2_: 0.8%).

**Table 2:** Participants’ reasons for voluntary blame-taking in Experiments 1 and 2.

	Reasons for blame-taking	
	Experiment 1	Experiment 2
	*n* (%)	*n* (%)
Greater consequences for the other person	104 (85.2)	56 (69.1)
I like the other person	95 (77.9)	47 (58.0)
The other person would do the same	80 (65.6)	44 (54.3)
I know the other person well	78 (63.9)	
I felt sorry for the other person	51 (41.8)	28 (34.6)
I am a good person	36 (29.5)	13 (16.0)
I felt pressured	8 (6.6)	3 (3.7)
The reward convinced me	1 (0.8)	1 (0.3)
The person is important to me		57 (70.4)
I can ask the person for a favor now		16 (19.8)
I wanted to help the other person		45 (55.6)
Other reasons	13 (10.7)	5 (6.2)

Empty cells occur when reasons were not provided as an answer option.

**Table 3:** Participants’ reasons against voluntary blame-taking in Experiments 1 and 2.

	Reasons against blame-taking	
	Experiment 1	Experiment 2
	*n* (%)	*n* (%)
I am an honest person	56 (62.9)	100 (66.2)
It was the other person’s own fault	44 (49.4)	53 (35.1)
I was too scared	24 (27.0)	14 (9.3)
The other person was not important to me	18 (20.2)	5 (3.3)
I felt too pressured	18 (20.2)	20 (13.2)
The other person would not do the same for me	16 (18.0)	24 (15.9)
The reward was not big enough	6 (6.7)	6 (4.0)
The consequences were too serious		25 (16.6)
The other person would not learn from her/his mistake		66 (43.7)
It is against the law		84 (55.6)
Other reasons	27 (30.3)	19 (12.6)

Empty cells occur when reasons were not provided as an answer option.

Most non-blame-takers indicated that they did not take the blame because they were *honest* (E_1_: 62.9%; E_2_: 66.2%). Few participants (E_1_: 6.7%; E_2_: 4.0%) indicated that *the reward was not big enough*. Other frequently named reasons were *it was the person’s own fault* (E_1_: 49.4%; E_2_: 35.1%) and because *it was against the law* (E_2_ only: 55.6%).

## Discussion

We tested the effect of relationship status and incentives on voluntary blame-taking in two experiments. A substantial number of participants were willing to take the blame for someone else (57.8 and 34.9%). In line with *Kinship Premium* and supporting Hypothesis 1b (but not Hypothesis 1a), relationship status moderated intended voluntary blame-taking. Participants imagining a close family member indicated more willingness to take the blame than participants imaging a close friend at the same level of emotional closeness (Rachlin and Jones, 2008). Unexpectedly and contrary to Experiment 1 and earlier findings (Willard et al., 2015, 2016; Willard and Burger, 2018), blame-taking did not differ significantly between the close friend and an acquaintance in Experiment 2. Contrary to Hypothesis 2, social incentives generally did not enhance intended blame-taking and only one blame-taker in each experiment indicated that the *incentive* had convinced them. This is in contrast to research showing that noncash incentives have a positive impact on prosocial behavior (Heyman and Ariely, 2004; Goette and Stutzer, 2008; Lacetera et al., 2014). Contrary to Hypothesis 3, we did not find an interaction effect between relationship status and incentives in both experiments. This contradicts the *Empathy-Altruism Hypothesis* (Batson, 1991). Reasons given for blame-taking suggest that factors other than an incentive may have a stronger impact on the decision to take the blame.

### Relationship Status and *Kinship Premium*

Our study tested the influence of kin relationships on intended blame-taking in an experimental setting. Participants took the blame significantly more often for close family members (84.3 and 52.9%) than for close friends (63.5 and 31.7%) at the same level of emotional closeness, suggesting that kin relationships play a crucial role when people consider whether to take the blame for someone else. These findings are in line with *Kin Selection Theory* and findings about a *Kinship Premium* in prosocial behavior (Rachlin and Jones, 2008; Curry et al., 2013; Pollet et al., 2013; Hackman et al., 2015). It seems that emotional closeness alone does not explain the increased blame-taking for close family members compared with close friends and that the information about relationship status has an independent effect beyond emotional closeness (Hackman et al., 2015). However, our experiments cannot explain which specific mechanisms are involved.

As expected, in Experiment 1, participants imagining a close friend more often took the blame than participants imagining an acquaintance. This is in line with the idea that emotional closeness is a key mediator of increased helping among non-kin and an important factor when people take the blame for someone else (Hackman et al., 2015; Willard et al., 2016; Willard and Burger, 2018). The result is also supported by the fact that a majority of blame-takers indicated that they would take the blame because they *liked the other person* (77.9%) and it is in line with previous experimental studies that found increased blame-taking for close friends compared to casual friends (Willard et al., 2016; Willard and Burger, 2018). However, we did not find a similar effect in Experiment 2. It could be that the difference in emotional closeness was not strong enough to have an impact on blame-taking. Indeed, participants in Experiment 2 felt substantially closer to the imagined acquaintances compared with participants in the first experiment (E_2_: *M* = 23.13 vs. E_1_: *M* = 17.15). The adapted description of an acquaintance in Experiment 2 may be the cause of this higher level of relationship closeness and might explain why we did not find a difference in blame-taking. Another reason could be that participants were younger in Experiment 1 (*M* = 26.01) than in Experiment 2 (*M* = 49.97). Half of the participants in Experiment 1 were 20 years old or younger. In contrast, half of the participants in Experiment 2 were 52 years old or younger and only 5% were 24 or younger. During young adulthood and adolescents, peer effects on risk taking and risky decisions are stronger than during adulthood (Gardner and Steinberg, 2005). Peer loyalty could have been an additional motivator to take the blame for close friends (Gudjonsson and Sigurdsson, 1994) and might explain the larger difference in blame-taking rates between close friends and acquaintances in Experiment 1 compared with Experiment 2.

### Incentives

In neither experiment, did we find an effect of social incentives on voluntary blame-taking. Following Experiment 1, we argued that participants might not have considered it a social reward when others hear about them falsely taking the blame. Indeed, taking the blame for a transgression might not be perceived as a “good deed” and might not display someone as a “nice person.” In Experiment 2, the social incentive constituted the person offering to cook the participant’s favorite meal as a thank-you. Again, we found no effect on blame-taking.

There are several potential reasons why we did not find an effect of social incentives. First, the social incentive might not have been big enough to affect the cost-benefit analysis and the benefit might not have exceeded the costs (Homans, 1961; Foa and Foa, 1976). Most studies that found positive effects of social incentives on prosocial behavior tested prosocial behavior that did not imply negative consequences for the helper (e.g., blood donation) and had a positive influence on self-image. Admitting to a traffic offense, on the other hand, might have a negative influence on self-image. Second, blame-taking involves a trust relationship because blame-takers usually know the other person. However, incentives can break social norms of trust and signal distrust (Gneezy et al., 2011). For other forms of prosocial behavior such as blood donations, where people contribute to public goods and trust relationships are not necessarily involved, incentives might have a stronger influence (Goette and Stutzer, 2008; Lacetera et al., 2014). Finally, it is possible that participants found it difficult to imagine the incentives and that they focused primarily on the person they had to imagine. This could also explain why neither social nor financial incentives affected blame-taking.

Neither did we find an interaction between relationship status and incentives on intended blame-taking in both experiments. However, when the costs are relatively low, most people are willing to help without expectations of repayment because they have a weak *communal* relationship with acquaintances and even strangers (e.g., calling an ambulance for a stranger or giving a stranger directions; Clark and Mills, 1993). It is possible that participants had a weak communal relationship with the imagined acquaintance and the incentives could have indicated distrust. Future research could test whether incentives increase blame-taking when there is absolutely no relationship between the blame-taker and the person in need that can be undermined by offering incentives. For example, German websites have a database of people (strangers) who are willing to take over an entry in the drivers’ registry for traffic violations in exchange for money.^2^

### Frequency of Self-Reported Blame-Taking

Participants in Experiment 1 reported higher willingness to take the blame than in Experiment 2 (E_1_: 57.8%; E_2_: 43.9%). Differences in the samples might offer an explanation for this finding. Participants in Experiment 1 were younger than participants in Experiment 2 (E_1_: *M_age_* = 26.01; E_2_: *M_age_* = 49.97) and were more often students (E_1_: 77.6%; E_2_: 2.2%). One possible reason for younger student participants to report taking the blame more often could be related to the traffic offense we used in our experiments. It is more likely that participants in Experiment 1 did not have a driver’s license or a car and committed fewer traffic offenses than participants in Experiment 2. As a result, they might not have had concerns about taking the blame for a traffic offense. Regardless, both experiments showed similar patterns of result regarding the influence of relationship status and incentives on self-reported blame-taking, allowing for some confidence in our conclusions, independent of blame-takers’ age. Future studies could ask whether participants have a driver’s license or test different blame-taking scenarios.

### Limitations and Future Directions

There are several limitations to this work. First, we used hypothetical scenarios where responses reflect willingness to take the blame but not actual blame-taking behavior. Participants did not sign a confession in front of an authority figure or had to actually carry the consequences. It could be that the blame-taking rate would be lower if participants actually had to admit to a transgression. One study that tested behavior found a voluntary blame-taking rate of 17% before interrogation (Pimentel et al., 2015). In this study, participants had the chance to take the blame for a confederate they had just known for the time of the experiment. This blame-taking rate is similar to the rate we found for acquaintances (23.9 and 23.2%). Second, we did not include romantic relationships in our study. However, research about prosocial behavior suggests that people help romantic partners and family members more often than close friends (Hackman et al., 2015) and in one self-report study several female prisoners indicated that they took the blame for their partners in order to protect them (Jones, 2011). To our knowledge, no experimental study has investigated voluntary blame-taking for romantic partners. Third, we used a minor traffic offense without criminal charges. It is likely that blame-taking would be substantially lower for more severe criminal offenses. However, an effect of relationship status would still be expected (Willard et al., 2016). Fourth, our participants may differ from typical crime suspects. Self-report studies found that blame-taking similar to false confessions is part of a criminal lifestyle (Gudjonsson et al., 2007). Therefore, one could expect an even higher blame-taking rate in a criminal population. Finally, participants in Experiments 1 and 2 indicated substantial differences in their demographics (e.g., mean age, differences in professions, and education level), which may have affected how they responded to the scenarios. Therefore, a comparison of the results must be made with caution.

## Conclusion

Although self-report studies suggest that voluntary blame-taking occurs frequently, few studies have investigated this phenomenon. Applying theories of prosocial behavior, the current study tested two possible situational risk factors for voluntary blame-taking. Our experiments contribute to the false confession literature and highlight the importance of kin relationships for decisions to take the blame for others. All in all, a substantial number of participants indicated their willingness to take the blame in two experiments. The blame-taking rate was enhanced for participants who previously had been in a similar blame-taking situation (60.7 and 46.7%). Our results are a first step to better understand under which circumstances people voluntarily take the blame for someone else and emphasize the importance of kin relationships.

## Data Availability Statement

Data of Experiment 1 and 2 can be found on Dataverse.NL and are available at: https://doi.org/10.34894/2H1ZBP.

## Ethics Statement

Ethical review and approval was not required for the study on human participants in accordance with the local legislation and institutional requirements. Written informed consent from the participants’ legal guardian/next of kin was not required to participate in this study in accordance with the national legislation and the institutional requirements.

## Author Contributions

TS, MS, and HM conceived the presented idea. TS and MS developed the theory. TS designed the experiment and analyzed the data. JC gave feedback on the first experiment and helped with MS to improve the method of the second experiment. TS took the lead in writing the manuscript with the support of MS. HM, JC, and JP contributed to the final version of the manuscript. All authors contributed to the article and approved the submitted version.

### Conflict of Interest

The authors declare that the research was conducted in the absence of any commercial or financial relationships that could be construed as a potential conflict of interest.

## Footnotes

^1^We used an attention check (“This is an attention check. Please click 1”) to check for data quality. Participants who did not pass the attention question were immediately excluded and were not able to finish the experiment.

^2^https://punkteloswerden.de/, https://flensburg-punkte-frei.de/
